# Retraction: Olanzapine-induced activation of hypothalamic astrocytes and toll-like receptor-4 signaling via endoplasmic reticulum stress were related to olanzapine-induced weight gain

**DOI:** 10.3389/fnins.2025.1713245

**Published:** 2025-10-03

**Authors:** Frontiers Editorial Office

The journal retracts the 13 January 2021 article cited above.

Following publication, concerns were raised regarding the integrity of the images in the published figures. The authors failed to provide the raw data or a satisfactory explanation during the investigation, which was conducted in accordance with Frontiers' policies. Given the concerns about the validity of the data, and the lack of raw data, the editors no longer have confidence in the findings presented in the article.

This retraction was approved by the Chief Executive Editor of Frontiers. The authors agreed to this retraction.

Frontiers would like to thank Dr. Sholto David for bringing the published article to our attention.

